# Cancer Outlier Analysis Based on Mixture Modeling of Gene Expression Data

**DOI:** 10.1155/2013/693901

**Published:** 2013-04-10

**Authors:** Keita Mori, Tomonori Oura, Hisashi Noma, Shigeyuki Matsui

**Affiliations:** ^1^Department of Statistical Science, School of Multidisciplinary Sciences, The Graduate University for Advanced Studies, 10-3 Midori-cho, Tachikawa, Tokyo 190-8562, Japan; ^2^Clinical Trial Coordination Office, Shizuoka Cancer Center, 1007 Shimonagakubo, Nagaizumi-cho Sunto-gun, Shizuoka 411-8777, Japan; ^3^Asia-Pacific Statistical Sciences, Lilly Research Laboratories Development Center of Excellence Asia Pacific, Eli Lilly Japan K. K. Sannomiya Plaza Building 7-1-5 Isogamidori, Chuo-ku, Kobe, Hyogo 651-0086, Japan; ^4^Department of Data Science, The Institute of Statistical Mathematics, 10-3 Midori-cho, Tachikawa, Tokyo 190-8562, Japan

## Abstract

Molecular heterogeneity of cancer, partially caused by various chromosomal aberrations or gene mutations, can yield substantial heterogeneity in gene expression profile in cancer samples. To detect cancer-related genes which are active only in a subset of cancer samples or cancer outliers, several methods have been proposed in the context of multiple testing. Such cancer outlier analyses will generally suffer from a serious lack of power, compared with the standard multiple testing setting where common activation of genes across all cancer samples is supposed. In this paper, we consider information sharing across genes and cancer samples, via a parametric normal mixture modeling of gene expression levels of cancer samples across genes after a standardization using the reference, normal sample data. A gene-based statistic for gene selection is developed on the basis of a posterior probability of cancer outlier for each cancer sample. Some efficiency improvement by using our method was demonstrated, even under settings with misspecified, heavy-tailed *t*-distributions. An application to a real dataset from hematologic malignancies is provided.

## 1. Introduction

Heterogeneity of the expression of oncogenes within the same histological cancers is considered to have significant implications for understanding disease biology, identifying risk groups, and optimizing patient treatment [1, 2]. Recently, Tomlins et al. [3] argued that traditional analytical methods, for example, a two-sample  *t*-statistic, which search for common activation of genes across a class of cancer samples, will fail to detect cancer genes which show differential expression in a subset of cancer samples or *cancer outliers*. They developed the “cancer outlier profile analysis” (COPA) method to detect cancer genes with such heterogeneous expression profiles within cancer samples and revealed subtypes of prostate cancer patients defined by recurrent chromosomal aberration.

Inspired by the COPA statistic, some authors have proposed other methods for detecting cancer-related genes with cancer outlier profiles in the framework of multiple testing [4–6]. However, such cancer outlier analyses will generally suffer from a serious lack of power because the analysis attempts to detect relatively small fractions of cancer outliers; the signal contained in the data is relatively limited, compared with that in the standard multiple testing setting where common activation of cancer-related genes for all cancer samples is supposed. As information sharing across units in the data generally improves efficiency of the analysis, we propose a simple efficient method via information sharing across both genes and cancer samples. Specifically, we propose a parametric normal mixture modeling of gene expression levels of cancer samples across genes after a standardization using the reference, normal sample data. Then, a gene-based statistic for gene selection is proposed on the basis of a posterior probability of cancer outlier for each cancer sample. This posterior probability itself is to provide a useful index to aid identifying cancer outliers for a selected gene. 

This paper is organized as follows. After providing a brief summary of the existing multiple testing methods for the cancer outlier analysis in Section 2, we provide the proposed method in Section 3. We assess performance of our methods via simulations in Section 4. An application to a real dataset from hematologic malignancies is given in Section 5. Finally, concluding remarks appear in Section 6. 

## 2. Existing Multiple Testing Methods for Cancer Outlier Analysis 

We suppose a microarray study to detect cancer-related genes from a large pool of *G* genes based on their gene expression levels measured for *n* samples, comprised of *n*_0_ samples from a normal class and *n*_1_ samples from a cancer class. The gene expression data considered here comprise normalized log ratios from two-color cDNA arrays or normalized log signals from oligonucleotide arrays (e.g., Affymetrix GeneChip). For gene *g*  (*g*  = 1,…, *G*), let *x*_*gi*_ be the expression value for sample *i* (*i* = 1, …, *n*_0_) in the normal class and let *y*_*gj*_ be that for sample *j* (*j* = 1,…, *n*_1_) in the cancer class. The most multiple testing methods developed for analyzing cancer outliers intend to a one-sided testing. Without loss of generality, we are interested in detection of activated genes that are overexpressed or upregulated in a subset of cancer samples, that is, cancer outliers. For detecting cancer-related genes with over- or underexpressions, one may perform two one-sided tests separately, one for detecting cancer-related genes with overexpressions and the other for detecting those with underexpressions.

The traditional two-sample  *t*-statistic for gene *g* is defined as

(1) $$\begin{array}{c} t_{g}=\frac{{\overset{-}{y}}_{g}-{\overset{-}{x}}_{g}}{s_{g}}, \end{array}$$

where ${\overset{-}{y}}_{g}$ is the mean expression value in the cancer samples, ${\overset{-}{x}}_{g}$ is the mean expression value in the normal samples, and *s*_*g*_ is the usual pooled standard error estimate for gene *g*  (*g* = 1,…, *G*). The  *t*-statistic is efficient in detecting cancer-related genes on which most cancer samples are activated, but may not be efficient for those with cancer outlier profiles. 

Tomlins et al. [3] defines the COPA statistic as

(2) $$\begin{array}{c} \text{Cop}{\text{a}}_{g}=\frac{q_{r}\left(y_{gj}:1\leq j\leq n_{1}\right)-\text{me}{\text{d}}_{g}}{\text{ma}{\text{d}}_{g}}, \end{array}$$

where  *q*_*r*_(·)  is the  *r*th percentile of the expression level, med_*g*_ is the median of expression values, and mad_*g*_ is the median absolute deviation of expression values in all of the samples:

(3) medgmedian(xgi,ygj;  i=1,…,n0,  j=1,…,n1),madg=1.4826×median(|xgi  −  medg|,|ygj−medg|;                 i=1,…,n0, j=1,…,n1).

The value of *r* in  *q*_*r*_(·), which represents a threshold in determining cancer outlier, is specified by the user, such as  *r* = 75, 90, or 95. 

Instead of using a fixed *r* percentile value, approximately equivalent to using the information from only one sample, the use of additional outlier samples can be more efficient. Specifically, the OS statistic [4] is defined as

(4) $$\begin{array}{c} \text{O}{\text{S}}_{g}=\frac{\sum_{i\in R_{g}}^{}\left(y_{gj}-\text{me}{\text{d}}_{g}\right)}{\text{ma}{\text{d}}_{g}}. \end{array}$$

Here the set of cancer outliers, *R*_*g*_, is heuristically identified by *R*_*g*_ = {*j* :  *y*_*gj*_ > *q*_75_(*x*_*gi*_, *y*_*gj*_ :  *i* = 1,…, *n*_0_;  *j* = 1,…, *n*_1_) + IQR(*x*_*gi*_, *y*_*gj*_ :  *i* = 1,…, *n*_0_;  *j* = 1,…, *n*_1_)}, where  IQR(*D*)  is the interquintile range of the data  *D*, IQR(*D*) = *q*_75_(*D*) − *q*_25_(*D*).

Wu [5] proposed the ORT statistic through identifying cancer outliers relative to the normal sample, rather than the pooled sample. Specifically, the ORT statistic is defined as

(5) $$\begin{array}{c} \text{OR}{\text{T}}_{g}=\frac{\sum_{i\in O_{g}}^{}\left(y_{gj}-\text{me}{\text{d}}_{g,x}\right)}{{\text{mad}}_{g}^{*}}, \end{array}$$

where *O*_*g*_ = {*j* : *y*_*gj*_ > *q*_75_(*x*_*gi*_ : *i* = 1,…, *n*_0_) + IQR(*x*_*gj*_ : *i* = 1,…, *n*_0_)},  med_*g*,*x*_ = median(*x*_*gi*_; *i* = 1,…, *n*_0_),  med_*g*,*y*_ = median(*y*_*gj*_; *j* = 1,…, *n*_1_), and

(6) madg∗=1.4826×median(|xgi−medg,x|,|ygj−medg,y|,               i=1,…,n0,  j=1,…,n1).

As the COPA, OS, and ORT statistics are criticized because the outliers are arbitrarily defined, Lian [6] considers all possible values of the outlier threshold. Specifically, for the ordered gene expressions for the cancer samples, ${\tilde{y}}_{g1}\geq {\tilde{y}}_{g2}\geq \cdots \geq {\tilde{y}}_{gn_{1}}$, the MOST statistic is defined as

(7) $$\begin{array}{c} \text{MOS}{\text{T}}_{g}=\underset{1\leq k\leq n_{1}}{\max}\left\{\frac{\left(\sum_{1\leq j\leq k}\left({\tilde{y}}_{gj}-\text{me}{\text{d}}_{g,x}\right)/{\text{mad}}_{g}^{*}\right)-\mu_{k}}{\sigma_{k}}\right\}, \end{array}$$

where *μ*_*k*_ = *E*⌊∑_1≤*j*≤*k*_ *z*_*j*_⌋ and *σ*_*k*_^2^ = Var⁡⌊∑_1≤*j*≤*k*_ *z*_*j*_⌋ for *z*_1_ > *z*_2_ > ⋯>*z*_*n*1_, the order statistics of *n*_1_ samples from the standard normal distribution. The standardization in the parenthesis is to make different values of the statistic comparable for different values of the outlier threshold, *k* (*k* = 1,…, *n*_1_).

## 3. The Proposed Method 

### 3.1. Mixture Modeling of Gene Expression Data

In order for information sharing across both genes and cancer samples, we propose a simple parametric normal mixture modeling of gene expression data of cancer samples. As the existing multiple testing methods, for each gene, we consider standardized gene expressions of the cancer samples based on the reference, normal sample data,

(8) $$\begin{array}{c} u_{gj}=\frac{y_{gj}-{\overset{-}{x}}_{g}}{s_{g,x}}, \end{array}$$

where *s*_*g*,*x*_ is the usual standard error estimate within the normal samples for gene *g*  (*g* = 1, …, *G*; *j* = 1, …, *n*_1_). Again, the standardization intends to make all gene expression data from the cancer samples comparable across genes. We then assume the finite normal mixture model with the three components,

(9) $$\begin{array}{c} f\left(u_{gj}\right)=\pi_{0}f_{0}\left(u_{gj}\right)+\pi_{1}f_{1}\left(u_{gj}\right)+\pi_{2}f_{2}\left(u_{gj}\right). \end{array}$$

The density function *f*_0_ corresponds to the null component with no differential expressions for the reference, normal sample data. The densities *f*_1_ and *f*_2_ correspond to the nonnull components (i.e., cancer outliers) of underexpression and overexpression, respectively, for the normal sample data. We specify normal distributions,  *N*(0, 1^2^),  *N*(*δ*_1_, 1^2^), and  *N*(*δ*_2_, 1^2^), for *f*_0_, *f*_1_, and *f*_2_, respectively. *π*_*k*_ represents the mixing proportion (*k* = 0, 1,2), and *π*_0_ + *π*_1_ + *π*_2_ = 1. We denote *Z*_*gj*,*k*_ as unobservable indicator random variables, such that *Z*_*gj*,*k*_ = 1 if the (standardized) expression level, *u*_*gj*_, of cancer sample *j* on gene *g* belongs to the  *k*th component, and *Z*_*gj*,*k*_ = 0 otherwise (*g* = 1,…, *G*; *j* = 1,…, *n*_1_). We estimate the parameters, *δ*_1_, *δ*_2_, and *π*'s, via applying the EM algorithm to cope with the unobservable indicator variable *Z*_*gj*,*k*_ in the mixture model (e.g., [7]). 

### 3.2. Statistics for Gene Selection

The posterior probability, *w*_*gj*,*k*_, that *Z*_*gj*,*k*_ = 1, that is, the expression level *u*_*gj*_ belongs to the  *k*th component, provides a basis for gene selection,

(10) $$\begin{array}{c} w_{gj,k}=\frac{{\hat{\pi }}_{k}{\hat{f}}_{k}\left(u_{gj}\right)}{\hat{f}\left(u_{gj}\right)}. \end{array}$$

For detecting overexpressed genes, possibly with a cancer outlier profile (as a one-sided testing), we propose to use the following gene-based statistic for gene selection:

(11) $$\begin{array}{c} S_{g}=1-\prod_{j=1}^{n_{1}}\left(1-w_{gj,2}\right). \end{array}$$

This statistic may correspond to one minus the posterior probability that none of samples are cancer outliers with overexpressions. We will select genes with greatest values of *S*_*g*_. Gene-based statistics for detecting underexpressed cancer-related genes can be similarly developed.

In our framework, we can also derive a similar gene-based statistic for detecting under- or overexpressed genes (as a two-sided testing). One has

(12) $$\begin{array}{c} T_{g}=1-\prod_{j=1}^{n_{1}}\left\{1-\left(w_{gj,1}+w_{gj,2}\right)\right\}. \end{array}$$

It is important to note that the posterior probabilities, *w*_*gj*,*k*_, themselves can serve as a helpful index to aid identifying cancer outlier samples for a particular (selected) gene. In contrast, the existing cancer outlier methods do not provide such an expression-level statistic for identifying cancer outlier samples. 

Unlike the existing statistics for cancer outlier analysis, the statistic, *S*_*g*_, does not involve any particular cancer outlier threshold, so that cancer-related genes with various proportions of cancer outliers (*ϕ* in Section 4), even those with common activation across all cancer samples, could be detected. However, as *S*_*g*_ is a composite of the posterior probabilities from all of the cancer samples, cancer-related genes with smaller proportions of cancer outlier will be more difficult to be detected because the statistic will be more dominated by the posterior probabilities from the cancer samples other than cancer outliers. The impact of the proportion of cancer outlier will be investigated in Section 4. 

## 4. Simulation Study

We conducted a simulation study to assess the performance of our method in detecting cancer-related genes with cancer outlier profiles. We considered a microarray study with  *G* = 10000  genes for *n* = 40, 80, or 200 samples, where the first half of samples were from the normal class and the latter half from the cancer class, that is, *n*_0_ = *n*_1_ = *n*/2. Of note, for a given *n*_1_, the power of the analysis will improve as *n*_0_ increases because more precise estimates of the mean and variance of the normal sample data become available in the standardization *u*_*gj*_ before fitting the mixture model (9) to detect cancer-related genes. We generated the gene expression levels for each gene from the standard normal distribution  *N*(0, 1^2^)  or the central  *t*-distribution with 20 degrees of freedom to assess the impact of deviation from the normality assumption. No interaction across genes was assumed. We supposed that *G* genes were divided into the three gene components according to the mixture model (9), that is, the null, underexpression, and overexpression component. The mixing proportions were set to as *π*_0_ = 0.6, *π*_1_ = *π*_2_ = 0.2. For each nonnull gene with under- or overexpressions, the proportion of cancer outliers in the cancer samples, *ϕ*, was set to be *ϕ* = 0.1, 0.3, or 0.5. We supposed a common difference or effect size in gene expression between the cancer outlier samples and the other samples (normal samples and nonoutlier cancer samples) across nonnull genes and set the value of *δ*_1_ as −2.0 and that of *δ*_2_ as 2.0. For each configuration, we performed gene selection based on the  *t*-statistic, COPA, OS, ORT, MOST, and the proposed one-sided statistics, *S*_*g*_, for detecting overexpressed cancer-related genes.

We assessed the false discovery rate (FDR) and true positive rate (TPR), defined as the proportion of false positives in the set of significant genes and the proportion of selected true positives in the set of all of the overexpressed genes ( = *Gπ*_2_), respectively. Note that the TPR corresponds to *average power* in multiple testing (e.g., [8, 9]). We conducted 200 simulations to obtain average TPR for a given value of FDR for each method, as the estimates of TPR were highly stable for *G* = 10000 values of each statistic obtained in a single simulation run. 

**Figure 1:**
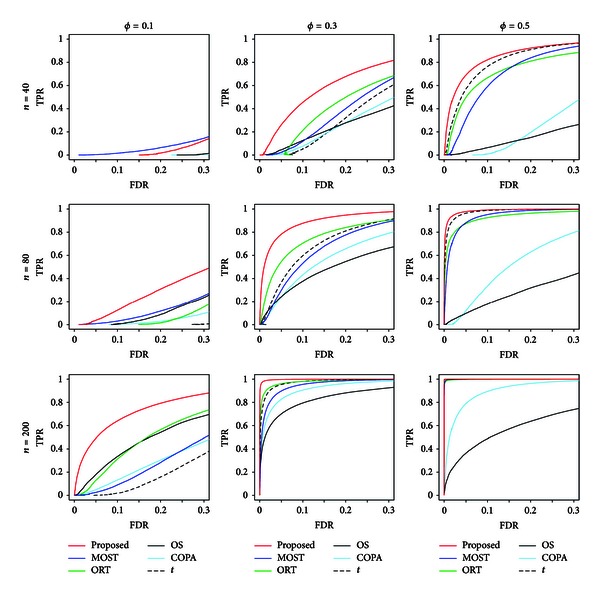
ROC curves that plot TPR versus FDR for normally distributed gene expression data.

**Figure 2:**
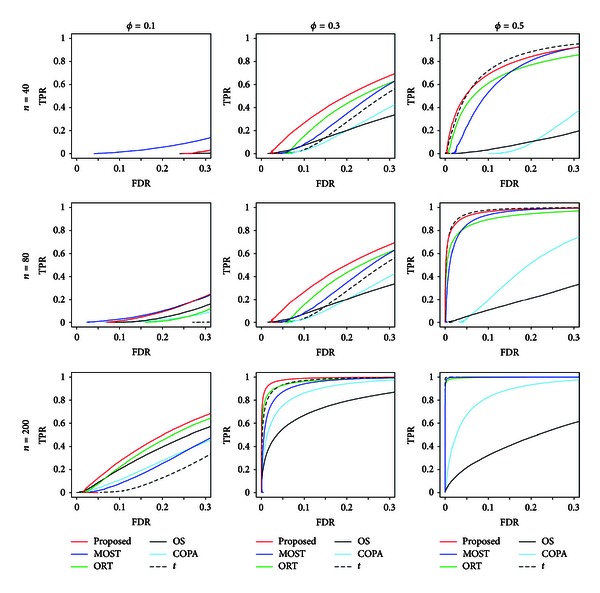
ROC curves that plot TPR versus FDR for  *t*-distributed gene expression data.

Figures 1 and 2 show ROC curves that plot the TPR and FDR for various numbers of significant genes in multiple testing for normally distributed and  *t*-distributed gene expressions, respectively. 

For normally distributed gene expressions, the gene selection based on the proposed statistic, *S*_*g*_, generally provided the greatest values of TPR (for a given value of FDR). As is expected, the proposed gene selection based on *S*_*g*_ provided greater TPR as *ϕ* increased. The gene selection based on the  *t*-statistic provided the smallest values of TPR, especially when the proportion of cancer outliers is small, such as *ϕ* = 0.1, but the TPR improved for greater values of the proportion, such as *ϕ* = 0.5, as is expected. The COPA and OS methods performed worst among the methods except the  *t*-test, especially for greater values of *ϕ*, such as *ϕ* = 0.5. In particular, the performance of the OS method was largely deteriorated for *ϕ* = 0.5. The ORT and MOST methods generally provided comparable TPR values, but less than those of the proposed method based on *S*_*g*_. 

For  *t*-distributed gene expressions, similar trends were observed. Again, the proposed method based on *S*_*g*_ provided greatest TPR in general, except for the scenario with *n* = 40 and *ϕ* = 0.1, although the degree of its superiority to the other methods, such as the ORT and MOST methods, becomes smaller, compared with the settings with normally distributed gene expressions. The COPA and OS methods again provided the smallest values of TPR, especially when *ϕ* is large, such as *ϕ* = 0.5. 

## 5. Application

We illustrate how the proposed method can capture the heterogeneity of cancer samples through its application to a microarray gene expression data from the myelodysplastic syndromes (MDSs) [10]. The MDSs are complex hematologic malignancies with heterogeneous clinicopathological features with various chromosomal aberrations. In order to discover the heterogeneous clinicopathological features of MDSs, possibly including those related to prognosis, we adopted the proposal using mixture distributions method for 139 MDSs and 69 nonleukemias (samples from bone marrow mononuclear cells from nonleukemic conditions), which were regarded as cancer and normal samples, respectively. Here, following Mills et al. [10], we removed samples of the chronic myelomonocytic leukemia disease type from MDS samples. We first adopted the RMA normalization [11] to the raw data (Raw data (CEL files) downloaded from Gene Expression Omnibus database (GEO, http://www.ncbi.nlm.nih.gov/geo/, accession number GSE15061)). We make statistics using the log scales expression intensities of each gene. As an initial screening of genes related to cancer outliers from a pool of  *G* = 54,675  candidate genes, we adopted the existing and proposal methods. For each method, we selected 200 top genes with the greatest values of the statistic. 

**Figure 3:**
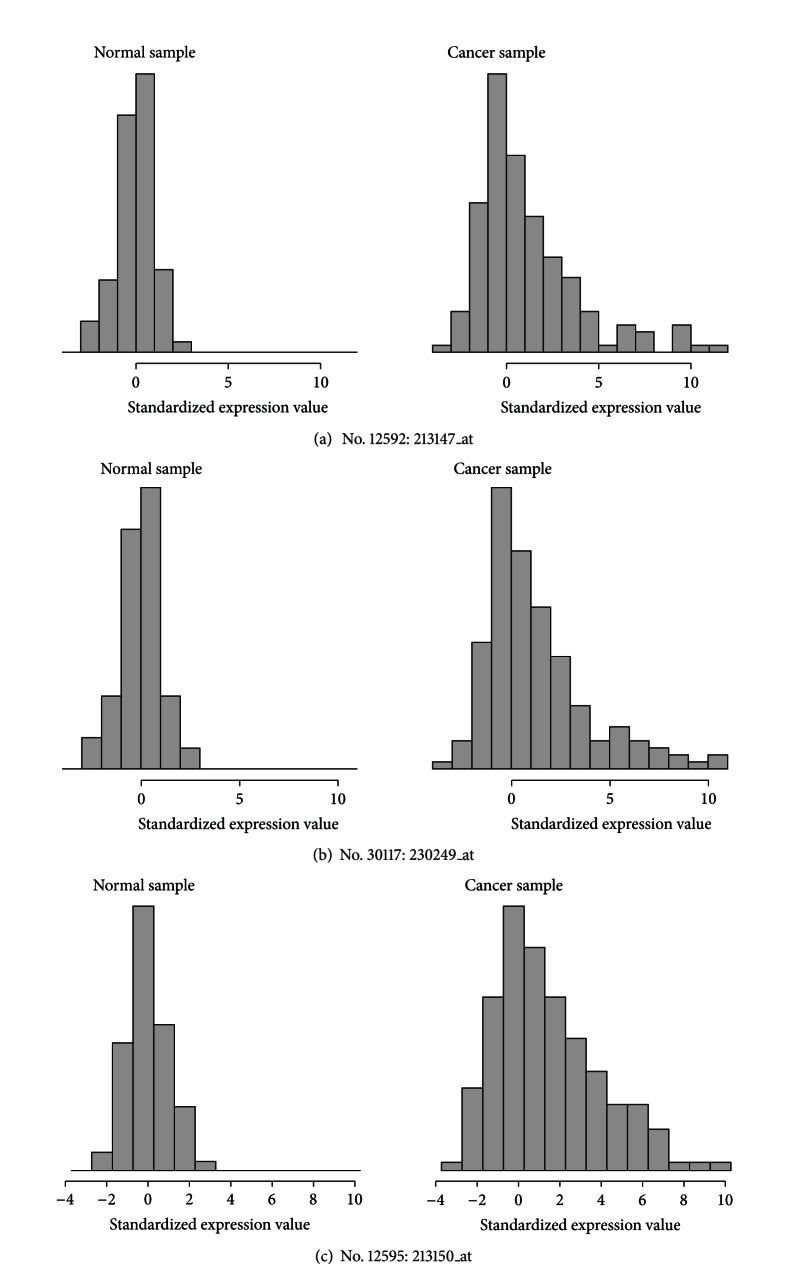
Histograms of the standardized expression values of three genes selected by our method, but not by the other methods.

The estimates of the parameters in the mixture model (9) obtained under an EM algorithm with a convergence criterion that are relative changes of the parameters <10^−4^ were as follows: ${\hat{\pi }}_{1}=0.018$, ${\hat{\pi }}_{2}=0.018$, ${\hat{\delta }}_{1}=-1.22$, and ${\hat{\delta }}_{2}=3.54$.  Table 1 summarizes the overlap in a number of selected genes between the gene selection methods. Generally, the OS, ORT, and MOST methods had substantial overlaps in the selected genes. The degree of overlap can be explained by the affinity among the methods in terms of the used standardization and outlier thresholds (see Section 2). On the other hand, it is interesting that the proposed method based on the gene-based statistic, *S*_*g*_, had intermediate overlaps with all of the other methods. This would indicate that the proposed method could detect cancer-related genes with various cancer outlier profiles.  Figure 3 shows histograms of the standardized expression levels within each class (normal and cancer) for three genes that were selected by our method, but not by the other methods. The proportion of cancer outliers was relatively small for the first two genes (Figures 3(a) and 3(b)), but large for the third gene (Figure 3(c)), which again indicates that our methods can detect cancer-related genes with various proportions of cancer outliers. 

## 6. Discussion

In this paper, we have attempted to improve the efficiency of the cancer outlier analysis through information sharing across genes and cancer samples. In our simulations, the proposed gene selection based on a parametric normal mixture modeling of gene expression data demonstrated some improvement in efficiency for detecting cancer-related genes with moderate to large proportions of cancer outlier (*ϕ* ≥ 0.3), even under settings with heavy-tailed  *t*-distributions. The proposed statistic would therefore be effective for selecting cancer-related genes that are involved in relatively major activation among cancer samples. Modification of the statistic for selecting cancer-related genes with more minor activation (i.e., small *ϕ*) is a subject for future research. Another important subject would be the addition of a gene-level mixture structure, that is, nonnull and null genes in terms of the association with cancer, to provide a more formal basis for evaluating false positives and true positives in gene selection. 

We have assumed the mixture structure (9) with the three components, *f*_0_, *f*_1_, and *f*_2_, that is common across genes. In some cases, the use of only one nonnull component for a particular direction of differential gene expression may be rather restrictive for plausible, large heterogeneity among cancer samples. Our method can be extended to involve multiple nonnull components, possibly with selection of the number of nonnull components based on model-selection criteria, such as AIC and BIC [7]. Another restriction of our model is that no interaction or correlation is assumed among genes. According to an investigation in the context of mixture modeling of a gene-level statistic (e.g., [12]), the impact of correlation is generally small for moderate correlation. In our modeling of the standardized gene expression levels *u*_*gj*_ across both genes and samples, the proportion of correlated *u*_*gj*_'s is expected to be relatively small because of independence across samples, but further investigation is needed. 

As to the existing methods of cancer outlier analysis, our simulations suggested superiority of the standardization based on the reference, normal sample data, not the pooled data from both cancer and normal samples. The poor performance of the OS method for greater proportions of cancer outliers, such as *ϕ* = 0.5, can be explained by the use of the IQR based on the pooled data. In such situations with relatively large numbers of cancer outliers, the IQR may cover some of cancer outliers, resulting in a very large outlier threshold, so that a substantial fraction of cancer outliers might be missed by using the statistic. In contrast, the performance of the ORT method, which is based on the IQR based only on the normal sample data, was not deteriorated as *ϕ* increased in our simulations. 

After screening cancer-related genes with cancer outlier profiles, researchers will need clustering of genes to identify coregulated genes that belong to the same molecular pathway related to disease biology and aggressiveness. At the same time, clustering of cancer samples based on the identified gene clusters can help discovering new taxonomy of cancer based on gene expression profiles of cancer outliers, possibly related to patients' clinical courses such as prognosis and response to therapeutics. A two-way model-based clustering of genes and samples in the context of cancer outlier analysis, as an extension of the proposed model-based method in this paper, would be an important topic, and one of such clustering methods will be reported elsewhere. 

## Figures and Tables

**Table 1:** The number of overlaps in selected genes between the gene selection methods in the example of hematologic malignancies. Top 200 genes were selected by each method.

	*t*-statistic	COPA	OS	ORT	MOST	Proposed
*t*-statistic	—	13	14	50	56	56
COPA	13	—	150	0	99	51
OS	14	150	—	139	108	86
ORT	50	0	139	—	151	89
MOST	56	99	108	151	—	75
Proposed	56	51	86	89	75	—
